# Prevalence, defect characteristics and risk factors associated with molar incisor hypomineralisation in Mexican schoolchildren: a cross-sectional study

**DOI:** 10.1007/s40368-025-01078-7

**Published:** 2025-07-16

**Authors:** M. Rivera, L. Karakowsky, C. E. Medina-Solís, M. de L. Márquez-Corona, D. J. Manton

**Affiliations:** 1 University Medical Center Groningen, Groningen, Netherlands; 2 Universidad Autónoma del Estado de Hidalgo, Pachuca, Mexico; 3 Universidad Tecnológica de México, Mexico City, Mexico; 4 Universidad Autónoma del Estado de México, Toluca, Mexico; 5 Academic Center for Dentistry Amsterdam, Amsterdam, Netherlands

**Keywords:** Molar incisor hypomineralisation, Prevalence, Associated factors, Distribution of MIH

## Abstract

**Background:**

Molar incisor hypomineralisation (MIH) is a developmental defect of decreased enamel mineral density, involving at least one first permanent molar and frequently, permanent incisors. The defects are demarcated and opaque, varying from white, yellow, and/or brown lesions to having post-eruptive enamel breakdown, with various clinical consequences. Mexican population studies have reported prevalence between 6.8 and 37.7%.

**Aim:**

To determine the prevalence, distribution and associated factors of MIH in Mexican schoolchildren aged 6–13 years from Pachuca, Mexico.

**Materials and methods:**

A cross-sectional study including 714 participants, selected randomly from nine public schools in Pachuca, Mexico. A dentist trained and standardised in the MIH-criteria of the European Academy of Paediatric Dentistry performed the clinical oral examinations, and questionnaires were developed to gather information regarding prenatal and perinatal health of the mother and child, as well as family sociodemographic and socioeconomic data.

**Results:**

The prevalence of MIH was 12.3% (95%CI 9.5–14.5%). The average age of schoolchildren with MIH was 9.1 ± 1.7 years, and without MIH was 8.7 ± 1.8 years; the mean number of affected first permanent molars was 2.4 ± 1.1 and 1.1 ± 1.4 for incisors. The factors statistically associated, age (OR = 2.46), fathers’ education (OR = 0.53), familial structure (OR = 4.69) and presence or absence of siblings (OR = 0.38).

**Conclusions:**

White demarcated opacities were the most prevalent type of defect, the severity of the lesions increased with the number of affected teeth. The factors associated with socioeconomic position were related to an increase in prevalence and severity.

## Introduction

Molar incisor hypomineralisation (MIH) is a prevalent, qualitative developmental enamel defect, occurring during amelogenesis, predominantly in the calcification or maturation phase (Bekes 2021; Weerheijm 2004). The definition of MIH is ‘at least one first permanent molar (FPM) having a demarcated opacity, with the permanent incisors commonly affected’, so MIH encompasses affected first permanent molars (FPMs) and permanent incisors (Weerheijm et al. 2001). The lesions present as demarcated opacities varying in colour from the most prevalent creamy white, to yellow and/or brown. The severity of the lesions increases with the number of affected teeth (Ghanim et al. 2011).

MIH-affected enamel has decreased mineral density, in conjunction with increased porosity and reduced hardness and elasticity, with a higher carbonate and protein content than the adjacent sound enamel (Crombie et al. 2013; Elhennawy et al. 2017). Due to the increased enamel porosity and weakened physical characteristics, post-eruptive breakdown (PEB) may occur in more severe (often yellow/brown) lesions, especially once lesions are exposed to occlusal forces; however, intact opacities are more prevalent than those with PEB (Crombie et al. 2013; Elhennawy et al. 2017).

The aetiological factors for MIH remain unclear, however, several risk factors have been associated with MIH, predominantly childhood illness within the perinatal and postnatal periods, with an over-riding genetic/epigenetic influence (Vieira and Manton 2019).

Oral health treatment burden is increased significantly by MIH, especially in more severe cases. It can affect the quality of life of individuals significantly due to dental pain and sensitivity, rapid development of carious lesions in those at increased caries risk, potential extraction and subsequent orthodontic consequences, and aesthetic problems (Jälevik 2010; Shields et al. 2024).

Therefore, the relevance of epidemiological research into the prevalence and characteristics of MIH in a given population is important. It can enhance public oral health, maximise efficient of use of available resources, and help ensure more specialised and effective care for MIH-affected individuals. In addition, the data elucidate the magnitude of the problem and the needs of the population with respect to health planning at the population level.

The worldwide prevalence is 12.8%, with wide regional variation from 0.48 to 46.6%, putatively due to study methodological variations such as varying age-groups, examination methods and validity, differing MIH indices and number of participants (Sluka et al. 2024). In Mexico, reported MIH prevalence ranges from 6.8 to 37.7%; however, the overall prevalence for Mexican children is reported to be between 15 and 20% (Medina Varela et al. 2024; Navarrete Esquivel 2016; Schwendicke et al. 2018).

The aim of the present study was to determine the prevalence of MIH in Mexican schoolchildren from Pachuca (population ≈ 650,000), describe intraoral location and characteristics of MIH-lesions and investigate associated risk factors.

As hypotheses, (1) the prevalence of MIH will be around 20% in the studied Mexican population; and (2) risk factors associated with the presence of MIH will be perinatal and postnatal health conditions of the child.

## Materials and methods

### Ethical issues

Ethical approval was obtained from the ethics and research committee of the Institute of Health Sciences of the Autonomous University of Hidalgo State (Comiteei.icsa/ICSa184/2022) from April 2023 to April 2024. Financial support was provided by Foundation Nakao for worldwide oral health, Lucerne, Switzerland, National Council of Humanities, Sciences and Technologies CONAHCYT, Mexico and University of Groningen, The Netherlands.

### Study design, setting and eligibility

A cross–sectional study was undertaken involving schoolchildren from nine of 122 randomly selected elementary public schools in the city of Pachuca, Mexico. The sample size calculation used the following parameters: confidence level of 95%, precision of 3%, estimated MIH prevalence of 20% based on an approximation of the mean prevalence in Mexico and reports from an area near the investigated city (Gurrusquieta et al. 2017; Irigoyen-Camacho et al. 2020), and 5% loss, which resulted in an estimated sample of 719 schoolchildren. Principals of the selected schools were contacted by the main researcher (MRP) and invited to participate; and depending on the number of students at each school and the sample size needed, a proportion from each school was chosen. The participants were selected randomly (http://www.randomizer.org) from a list of names provided by the schools, and prior to the clinical examination a total of 1000 informed consent forms and questionnaires were delivered to the relevant parents.

### Inclusion, exclusion and elimination criteria

The inclusion criteria were female and male children from 6 to 13 years-of-age studying at the elementary level in public schools and an informed consent signed by parents/guardians and assent from the participant. The exclusion criteria were children who had a disability that prevented oral examination, a syndrome/condition that potentially affected the oral cavity, children who did not have consent for the oral examination and participants/parents who did not complete the questionnaire fully (fewer than 75% of questions answered clearly).

### Variables and information collection

Prior to conducting the study, training and calibration for MIH epidemiological studies based on the criteria proposed by the European Academy of Paediatric Dentistry (EAPD) was carried out for the main researcher (MRP) with an experienced trained and calibrated examiner (DJM) at the University of Groningen, The Netherlands (Kappa > 0.80).

The questionnaires were developed to gather information regarding prenatal and perinatal health of the mother and child, as well as sociodemographic and socioeconomic data of the family (Ghanim et al. 2013; Whatling and Fearne 2008), which were given to the parents before the clinical examination, along with the informed consent form. Parents completed the questionnaires at home and after a period of 3–5 days, returned the completed questionnaires and signed consent forms.

The oral examinations were carried out by MRP and two assistants in a designated area of the school equipped with tables and chairs, and in some schools an observer who was either a teacher or a volunteer parent was present. To facilitate the cooperation of the children, chairs were placed facing each other, so that whilst an oral examination was in progress, the next child to be examined was seated in the adjacent chair observing the process which putatively helped behaviour, applying the modelling technique.

With provided age-appropriate toothbrushes and toothpaste containing 1450 ppm sodium fluoride (“Triple acción”, Colgate-Palmolive Company, NY, USA.), participants brushed their teeth before the examination under the supervision of the examiner or an assistant. The examiner used an artificial white light lamp (high-performance LED headlamp) to illuminate the oral cavity. The tooth surfaces were dried with sterile cotton rolls, and a WHO dental probe with a round tip and plane mouth mirror were used to examine the teeth. Tables and chairs were cleaned with ethanol—alkyl dimethyl benzyl ammonium saccharinate (Lysol, Reckitt, Berkshire, UK) between participants.

The presence or absence of MIH was determined based on the EAPD criteria (Table 1) and after the oral examination the child’s MIH status was reported to the parents through a written report (Ghanim et al. 2015). To determine if participants with MIH had hypersensitivity of MIH-affected teeth, the examiner rated the response to stimulation using the Schiff Cold Air Sensitivity Scale (SCASS), using a triplex syringe, a jet of air for 1 s at a distance of 1 cm and perpendicular to the occlusal surface of the affected tooth; neighbouring teeth were shielded using cotton rolls.

**Table 1:** MIH criteria of the EAPD

Code	Description
0	No visible enamel defect
1	Enamel defect, not MIH/HSPM
11	Diffuse opacity
12	Hypoplasia
13	Amelogenesis imperfecta
14	Hypomineralisation defect (not MIH/HSPM)
2	White, creamy demarcated, yellow or brown demarcated opacities
21	White or creamy demarcated opacities
22	Yellow or brown demarcated opacities
3	Post–eruptive enamel breakdown (PEB)
4	Atypical restoration
5	Atypical caries
6	Missing due to MIH/HSPM
7	Cannot be scored
Lesion extension criteria (index teeth only, scores 2–5)	
I	Less than one third of the tooth affected
II	At least one third but less than two thirds of the tooth affected
III	At least two thirds of the tooth affected

A defect of < 1 mm in diameter was considered as sound enamel

The dependent variable was the presence or absence of MIH. The independent variables were child sex, age, maternal education, paternal education, family structure, type of family housing, reported pregnancy complications (including arterial hypotension, arterial hypertension, toxoplasmosis, pre-eclampsia, psychological stress), caesarean delivery, perinatal complications (jaundice, neonatal hypoxia, respiratory diseases, hypocalcaemia), pregnancy medication, breastfeeding, formula milk, childhood diseases (including ear infections, tonsillitis, pneumonia, asthma, bronchitis, influenza, high fever, severe gastrointestinal infection, severe urinary tract infection, measles, kidney disease, chicken pox, dental trauma), age-related vaccines received by the child according to Mexico's health policies, antimicrobial medication and ingestion of vitamin supplements and fluoride supplements during the first 3 years-of-life.

### Statistical methods

Data were analysed using Stata software (Stata Corp., CA, USA). Initially, descriptive analyses were performed—frequencies and percentages were calculated for qualitative variables and means and standard deviations calculated for quantitative variables. Chi squared or Fisher-Exact tests were used for nominal or ordinal variables to examine the association between selected variables and the prevalence of MIH, and odd ratios (OR) with a 95% confidence interval (95% CI) were calculated to determine the association of age and the presence or absence of MIH. A *p* value of < 0.05 was considered statistically significant.

## Results

Of the 1000 questionnaires distributed, 721 were returned signed, seven were excluded due to incomplete recorded information leaving a total of 714 participants (71% response rate). The mean participant age was 8.7 ± 1.8 years; 371 (52%) were female. The mean age for female were 8.8 ± 1.8 and for male 8.6 ± 1.8. Age was categorised into two groups, 6 to 8 years with 320 participants (44.8%) and 9 to 13 years, 394 participants (55.2%).

**Fig. 1:**
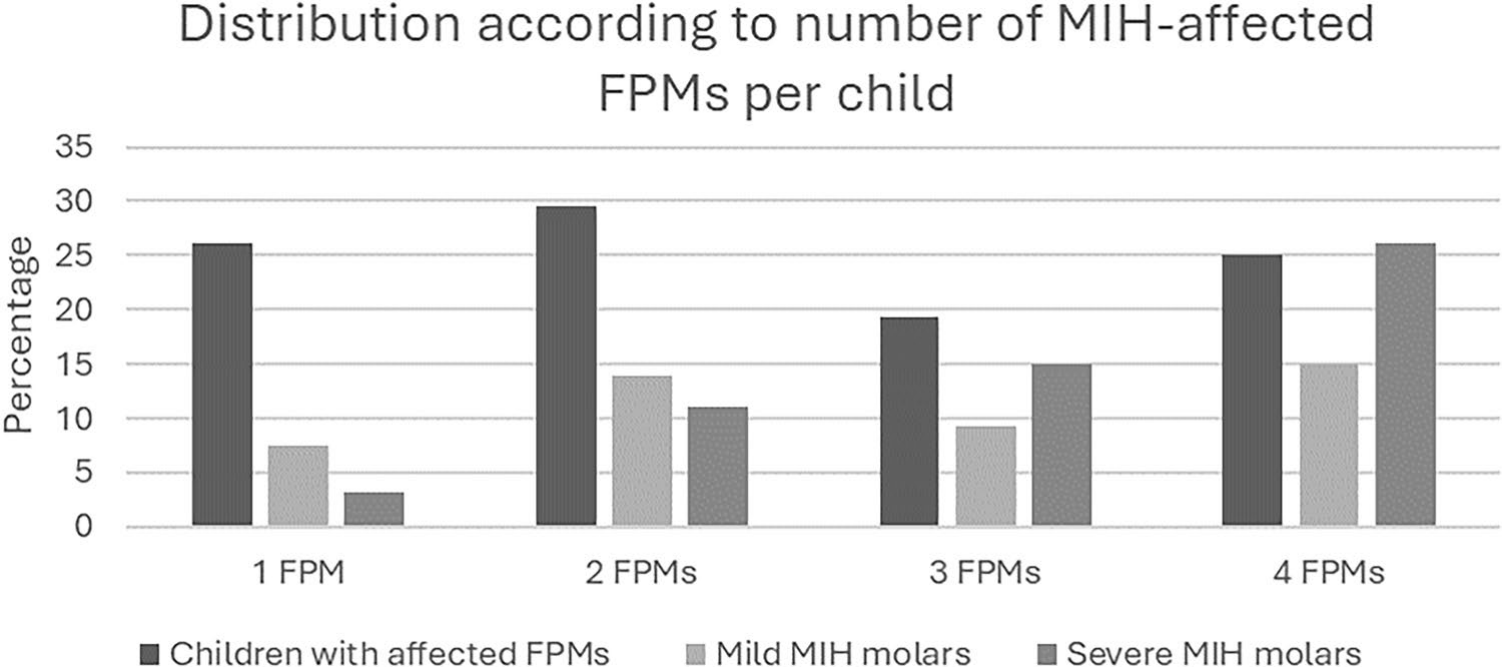
Overall distribution (%) of MIH-affected first permanent molars per child, according to number of affected FPMs and severity

Molar incisor hypomineralisation was present in 88 participants (12.3%; 95% CI 9.5–14.5%). Mean participant age with MIH was 9.1 ± 1.7 years, female with MIH 9.1 ± 1.7, male with MIH 9.0 ± 1.7 and without MIH 8.7 ± 1.8 years. In MIH-affected individuals, the average number of MIH-affected FPMs was 2.4 ± 1.1, of whom 46 (52%) also had MIH-affected anterior teeth (1.1 ± 1.4). Twenty-seven (30.6%) participants had dental sensitivity in at least one FPM. The number of MIH-affected FPMs per child according to severity is illustrated in Fig. 1; 23 (26.1%) children had one FPM affected, 17 (19.3%) had two, 26 (29.5%) had three FPMs affected, and 22 (25%) had four FPMs affected. Molars with opacities only (codes 21 and 22) were defined as mild, whereas post-eruptive breakdown, atypical caries/restoration or missing due to MIH (codes 3–6) were classified as severe.

**Fig. 2:**
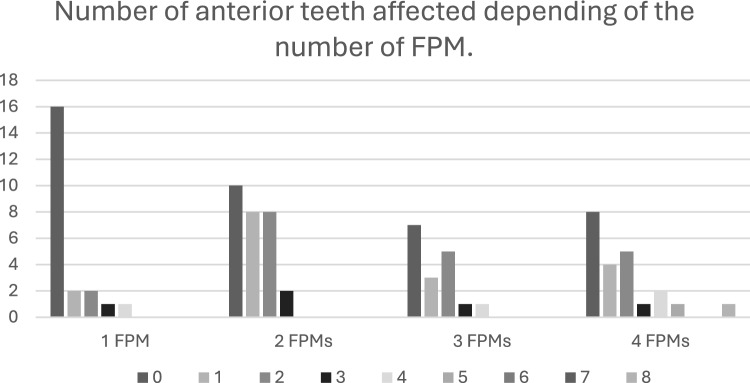
Comparison between the number of anterior affected teeth and the number of FPM

The relationship between the number of affected anterior teeth and the number of FPM with MIH is illustrated in Fig. 2. Participants with one, two or three FPMS affected exhibited up to four anterior teeth with hypomineralised lesions, those with four FPM affected showed a broader range, with some individuals having up to eight anterior teeth affected.

The distribution characteristics of MIH in the FPMs is tabulated in Table 2. The total number of MIH-affected FPMs was 214, including 78 (36.4%) white or creamy lesions and 16 (7.5%) yellow or brown lesions; 9 (4.2%) exhibiting PEB, 22 (10.3) atypical restorations, 78 (36.4%) having atypical carious lesions. Regarding the total number of affected permanent anterior teeth (*N* = 99); two (2%) had PEB, 77 (77.8%) had white or creamy lesions, 17 (17.2%) were yellow or brown and atypical caries lesions occurred in three teeth (3%).

**Table 2:** Categorisation of the characteristics of MIH-affected first permanent molars

Tooth	Code 21 *N* (%)	Code 22 *N* (%)	Code 3 *N* (%)	Code 4 *N* (%)	Code 5 *N* (%)	Code 6 *N* (%)	Code 7 *N* (%)	Total *N* (%)
16	23 (10.7)	6 (2.8)	1 (0.5)	0	6 (2.8)	2 (0.9)	3 (1.4)	41 (19.1)
26	18 (8.4)	7 (3.2)	1 (0.5)	0	20 (9.3)	0	0	46 (21.5)
36	17 (7.9)	1 (0.5)	4 (1.8)	17 (7.9)	22 (10.3)	1 (0.5)	1 (0.5)	63 (29.4)
46	20 (9.3)	2 (0.9)	3 (1.4)	5 (2.3)	30 (14.0)	2 (0.9)	2 (0.9)	64 (29.9)
Total	78 (36.4)	16 (7.5)	9 (4.2)	22 (10.3)	78 (36.4)	5(2.3)	6 (2.8)	214 (100)

*Code 21* white or creamy demarcated opacities, *Code 22* yellow or brown demarcated opacities, *Code 3* post-eruptive enamel breakdown (PEB), *Code 4* atypical restoration, *Code 5* atypical caries lesion, *Code 6* missing due to MIH/HSPM, *Code 7* cannot be scored

The distribution and prevalence of hypomineralised defects are summarised in Table 3; 49 (55.7%) females had MIH, compared to 39 (44.3%) males, and 28 (31.8%) children in the 6–8 year age group and 60 (68.2%) children in the 9–13 year age group had MIH, equivalent to 3.9% and 8.4% prevalence, respectively.

**Table 3:** Distribution and prevalence of hypomineralised defects by sex and age

MIH-affected teeth	Total *N* (%)	Female *N* (%)	Male *N* (%)	Age 6–8 y *N* (%)	Age 9–13 y *N* (%)
One incisor	16 (18.2)	11 (22.4)	5 (12.8)	2 (7.1)	14 (23.3)
> One incisor	30 (34)	18 (36.7)	12 (30.8)	8 (28.6)	22 (36.7)
One FPM	23 (26.1)	12 (24.5)	11 (28.2)	9 (32.1)	14 (23.3)
> One FPM	65 (73.9)	37 (75.5)	28 (71.8)	19 (67.8)	46 (76.7)
FPM and incisors	46 (52.3)	29 (59.2)	17 (43.6)	10 (35.7)	36 (60)
Number of children with MIH N (%)	88 (100)	49 (55.7)	39 (44.3)	28 (31.8)	60 (68.2)

Demarcated hypomineralised lesions affecting other permanent teeth (HOPT) were present in 34 teeth in 23 children from the 563 participants (4.1%) with at least one fully erupted canine or first premolar or second premolar or second permanent molar; 6 (26%) of these participants also had MIH.

In the HOPT assessment, amongst the 161 participants with fully erupted permanent canines, only four (2.5%) exhibited signs of demarcated opacities. Amongst the 221 individuals with fully erupted first premolars, 12 (5.4%) children had demarcated opacities. In the case of second premolars, 14 out of 140 participants (10%) had demarcated opacities, and four (9.7%) of the 41 participants with fully erupted second permanent molars had demarcated opacities.

Regarding maternal education, 461 (64.6%) mothers studied further than high school level compared to 390 (54.6%) fathers. The family structure for participants was that 620 (86.8%) participants had two parents ‘at home’ and 94 (13.2%) were cared for by their mothers; 394 (55.2%) families owned their house, whilst 216 (30.2%) rented their house (Table 4).

**Table 4:** Bivariate analysis of the associations between MIH and sociodemographic, peri- and post-natal health conditions of mother and child

Variables	Without MIH *N* (%)	With MIH *N* (%)	*X*^2^-value *p*-value
Sex			
Female	322 (86.8)	49 (13.2)	*X*^2^ = 0.5568
Male	304 (88.6)	39 (11.4)	*p* = 0.456
Mother’s education			
Elementary and secondary school	216 (85.4)	37 (14.6)	*X*^2^ = 1.9176
High school or further	410 (88.9)	51 (11.1)	*p* = 0.166
Father’s education			
Elementary and secondary school	275 (84.9)	49 (15.1)	*X*^2^ = 4.2991
High school or further	351 (90.0)	39 (10.0)	*p* = 0.038
Familiar structure			
Single parent	86 (91.5)	8 (8.5)	*X*^2^ = 1.4575
Two parents	540 (87.1)	80 (12.9)	*p* = 0.227
Child—siblings			
Single child	77 (75.5)	25 (24.5)	*X*^2^ = 11.5276
Siblings	463 (88.2)	62 (11.8)	*p* = 0.001
Housing			
Loaned house	91 (87.5)	13 (12.5)	*X*^2^ = 1415
Rented	188 (87.0)	28 (13.0)	*p* = 0.932
Owned	347 (88.0)	47 (12.0)	*X*^2^ = 1415
Pregnancy illnesses			
None	510 (86.7)	78 (13.3)	*X*^2^ = 2.7268
At least one	116 (92.0)	10 (8.0)	*p* = 0.099
Delivery complications			
None	609 (87.8)	85 (12.2)	*X*^2^ = 0.1363
At least one	17 (85.0)	3 (15.0)	*p* = 0.712
Caesarean delivery			
No	412 (87.9)	57 (12.1)	*X*^2^ = 0.0372
Yes	214 (87.4)	31 (12.6)	*p* = 0.847
Medication during pregnancy			
No	436 (87.2)	64 (12.8)	*X*^2^ = 0.3484
Yes	190 (88.8)	24 (11.2)	*p* = 0.555
Smoking during pregnancy			
No	614 (87.8)	85 (12.2)	*X*^2^ = 2.8862
Yes	7 (70.0)	3 (30.0)	*p* = 0.089
Prematurity (less than 37 weeks)			
No	499 (86.9)	75 (13.1)	*X*^2^ = 1.4886
Yes	127 (90.7)	13 (9.3)	*p* = 0.222
Breastfeeding			
No	80 (90.9)	8 (9.1)	*X*^2^ = 0.9715
Yes	546 (87.2)	80 (12.8)	*p* = 0.324
Formula milk			
No	162 (83.9)	31 (16.1)	*X*^2^ = 2.7064
Yes	435 (88.6)	56 (11.4)	*p* = 0.100
Soy extract			
No	431(86.5)	67 (13.5) 20	*X*^2^ = 1.1132
Yes	171(89.5)	(10.5)	*p* = 0.291
Childhood illnesses during the first three years of age			
Otitis media			
No	583 (87.3)	85 (12.7)	*X*^2^ = 0.5238
Yes	32 (91.4)	3 (8.57)	*p* = 0.469
Tonsillitis			
No	554 (88.2)	74 (11.8)	*X*^2^ = 1.8691
Yes	68 (82.9)	14 (17.1)	*p* = 0.172
Pneumonia			
No	596(87.6)	84 (12.4)	*X*^2^ = 0.0254
Yes	26 (86.7)	4 (13.3)	*p* = 0.873
Asthma			
No	608 (87.4)	88 (12.6)	*X*^2^ = 2.0205
Yes	14 (100.0)	0	*p* = 0.155
Bronchitis			
No	586 (87.6)	83 (12.4)	*X*^2^ = 0.0016
Yes	36 (87.8)	5 (12.2)	*p* = 0.968
Influenza			
No	599 (87.5)	86 (12.5)	*X*^2^ = 0.3801
Yes	22 (91.7)	2 (8.3)	*p* = 0.538
High fever			
No	471 (86.9)	71 (13.1)	*X*^2^ = 1.0013
Yes	150 (89.8)	17 (10.2)	*p* = 0.317
Severe gastrointestinal infection^a^			
No	583 (87.5)	83 (12.5)	*X*^2^ = 0.0459
Yes	39 (88.6)	5(11.4)	*p* = 0.830
Childhood illness			
None	356 (88.8)	45 (11.2)	*X*^2^ = 1.0299
At least one illness	270 (86.3)	43 (13.7)	*p* = 0.310
Dental trauma			
No	608 (87.7)	85 (12.3)	*X*^2^ = 0.4426
Yes	14 (82.4)	3 (17.6)	*p* = 0.506
Antimicrobials for child			
No	259 (86.9)	39 (13.1)	*X*^2^ = 0.27510
Yes	367 (88.2)	49 (11.8)	*p* = 0.60
Familial history of ‘weak teeth’			
No	398 (86.5)	62 (13.5)	*X*^2^ = 1.5917
Yes	228 (89.8)	26 (10.2)	*p* = 0.207
All childhood vaccines			
No	33 (86.8)	5 (13.2)	*X*^2^ = 0.0186
Yes	586 (87.6)	83 (12.4)	*p* = 0.891
General anaesthesia during the first 3 years of life			
No	569 (87.0)	85 (13.0)	*X*^2^ = 1.7473
Yes	44 (93.6)	3 (6.4)	*p* = 0.186
Vitamins			
No	470 (88.5)	61 (11.5)	*X*^2^ = 1.3437
Yes	156 (85.3)	27 (14.7)	*p* = 0.246

^a^Severity of illness was determined by parent(s)

For maternal illnesses during pregnancy, 588 (82.5%) mothers had no reported illness and 126 (17.6%) had at least one illness; 699 (98.6%) of the mothers reported not having smoked during pregnancy, whilst 10 (1.4%) reported smoking one or two cigarettes per day. Of the smoking mothers, three of ten had children with MIH (30%). Caesarean section delivery was undertaken for 245 (34.3%) participants and 626 (87.7%) children were breastfed at some point of infancy; and 612 (83.7%) had siblings. Formula milk was used to feed 491 (71.8%) of the participants and 191 (27.7%) had soy extract as part of their diet at some time point within the first two years-of-life (Table 4).

During the first 3 years-of-life, 313 (43.8%) of the children had at least one reported illness. One-hundred-and-forty-six (58.3%) children had taken antibiotics such as penicillin, amoxicillin or a cephalosporin. Regarding tooth brushing frequency of the participants, 166 (23.2%) brushed once-per-day, 403 (56.5%) twice, and 145 (20.3%) three times.

A bivariate analysis of all the parental variables indicated that sociodemographic factors such as father’s education (elementary and secondary school–high school or further), familial structure, whether the participant was an only child or had siblings, and age were statistically significantly associated with the presence of MIH (Table 4).

A multivariate logistic regression analysis of variables associated with the presence of MIH, indicated that child age, father´s education, familial structure and presence or absence of siblings were statistically significant associated with MIH (Table 5). The child age variable indicated that MIH was 2.46 times more frequent in children between the ages of 9 and 13 years old compared to those aged 6 to 8 years.

**Table 5:** Multivariate analysis of associations between MIH and bivariate statistically significant variables

Variable	OR (95% CI)	*p* value
Sex		
Female	1*	0.885
Male	0.96 (0.56–1.64)	
Age		
6–8 years old	1*	0.003
9–13 years old	2.46 (1.36–4.45)	
Father’s education		
Elementary and secondary school	1*	0.025
High school or further	0.53 (0.31–0.92)	
Familiar structure		
Single parent	1*	0.012
Two parents	4.69 (1.39–15.72)	
Child—siblings		
Single child	1*	0.003
Siblings	0.38 (0.20–0.73)	

*OR* odds ratio, *CI* confidence of interval, * reference category

The odds of MIH being present in children whose fathers had an education level of high school or further (OR = 0.53; *p* = 0.025) and the presence of siblings (OR = 0.38; *p* = 0.003) was significantly decreased; conversely, the odds of MIH being present in children whose familial structure included both parents was significantly increased (OR = 4.69; *p* = 0.012).

## Discussion

The present study represents the first investigation of MIH in Pachuca, Mexico; determining a prevalence of 12.3%, aligning with the 13.5% mean MIH prevalence reported in a 2021 systematic review of Mexican populations (Lopes et al. 2021). However, reported Mexican MIH prevalence varies widely from 6.8% to 37.7% (Medina Varela et al. 2024; Navarrete Esquivel 2016), reflecting the global variability in MIH prevalence values, which range from 0.48 to 46.6% (Sluka et al. 2024). The differences in diagnostic criteria, examiner experience, sampling methods (random, convenience, stratified, etc.) and socioeconomic and geographical factors such as age, gender, social position, access to healthcare for mother and child and geographical location putatively contribute to these discrepancies (Schwendicke et al. 2018; Sluka et al. 2024). The studies conducted in school settings or dental clinics may show differences in prevalence because variability in the ability of the population to access and utilise educational and/or oral health services and create a sample bias.

Demarcated hypomineralised lesions in other permanent teeth (HOPT) were detected in 23 participants (4.1%); being the second premolar most commonly affected, which contrasts with a Greek study of 1156 adolescents with 23% HOPT prevalence, predominantly affecting the second permanent molars (Kevrekidou et al. 2021). These differences emphasise the difficulties in comparing different age-related cohorts, as the present study included children aged 6–13 years limiting the number of permanent non-MIH index teeth present, compared to 14 year-olds in the Greek study where most participants would have erupted canines, second permanent molars and premolars.

Approximately half of the present participants with MIH had affected permanent incisors, compared to 36.6% in a recent systematic review (Lopes et al. 2021), which was outside the upper 95% CI of 43.7%; although several studies have reported anterior prevalence > 50%. Amongst MIH-affected FPMs, 30.6% exhibited hypersensitivity/pain, similar to the 34.7% reported in 8 year-olds in Brazil (Raposo et al. 2019), however, an even higher prevalence (45%) of hypersensitivity/pain was reported in a systematic review (Santos et al. 2024); indicating that MIH-associated pain and discomfort is a prevalent issue. Sensitivity is complex to manage successfully, with limited supporting evidence for desensitising interventions (Cavalcante et al. 2024; Hjertberg et al. 2024). Hypersensitivity can significantly impact oral hygiene practices in dental quadrants with MIH-affected FPMs, as children may avoid brushing due to discomfort, increasing their susceptibility to the development of caries lesions (in those at increased caries risk), post-eruptive breakdown and subsequent restorative care, which often has poor outcomes (Raposo et al. 2019). Given this high prevalence of hypersensitivity/pain and association with poor outcomes, oral health clinicians must prioritise early efforts to limit sensitivity via proactive intervention strategies, such as decreasing caries risk, increasing mineral gain and preventing PEB, especially in severely affected teeth, however, how to achieve this is still debatable (Cavalcante et al. 2024; Hjertberg et al. 2024).

Demarcated lesions without PEB were the most common type of defect, with FPMs displaying a higher proportion of white/creamy defects compared to yellow or brown opacities. Notably, as the number of molars affected by MIH increased, lesion severity also escalated, and the number of anterior teeth affected likewise increased, echoing previous reports (Ghanim et al. 2011; Gurrusquieta et al. 2017; Sosa-Soto et al. 2022). This pattern strongly suggests a ‘dose dependent’ relationship, the greater the exposure to the aetiological factor(s) of MIH, the more teeth are affected, and the more severe the defects become (Lygidakis et al. 2008).

There was no statistically significant difference in MIH prevalence between maxillary and mandibular FPMs, with conflicting existing evidence (Abdalla et al. 2021; Ghanim et al. 2011; Preusser et al. 2007). Interestingly, in the present study, mandibular MIH-affected FPMs exhibited a higher prevalence of carious lesions, restorations and PEB, potentially due to their earlier eruption, leading to greater time exposure to occlusal forces and cariogenic environments (when present) (Wuollet et al. 2018).

Maxillary permanent incisors had a significantly higher MIH lesion prevalence than mandibular incisors, and like the FPMs, white/creamy demarcated lesions were the most common finding in incisors, consistent with other studies (Almuallem et al. 2022; Altan and Yilmaz 2023; Ghanim et al. 2011).

A notable finding on the present study is the association of MIH with father’s education level, familial structure, presence of siblings and child age. Higher socioeconomic position (SEP) appeared to be a protective factor against MIH; lower SEP may increase MIH risk and severity potentially due to increased childhood illness, nutritional deficiencies and limited healthcare access, factors that are particularly relevant across Latin America, Brazil, Chile, and Mexico (Franco et al. 2024; Harz et al. 2023; Rai et al. 2018). However, the relationship between SEP and MIH is multifactorial and highly complex, with conflicting evidence across studies (Villanueva-Gutiérrez et al. 2019; Wuollet et al. 2018). The presence of siblings was identified as a protective factor against MIH in the present study, a finding that contrasts with research conducted in an Iraqi population, where a higher number of children in a family correlated with an increased prevalence of MIH (Ghanim et al. 2013). In the present study, the largest family had four children, with families of this size being infrequent. Conversely, in the Iraqi study, families had up to nine children, with four to six being the most common. In Mexico, family size has been decreasing over time, however, there is still an influence of SEP on family size, with families with lower socioeconomic income tending to have more children (Villasmil 2022). Family size in 1976 was reported to have an average of 4.8 members, in 1997, 3.1 and in 2006 it was reduced to 2.7 members (Menkes and Mojarro 2006), which may be the reason why the results of this study differ from those mentioned above in the Iraqi population.

The age of the participants was associated with higher prevalence of MIH, potentially due to the mean age being between 8 and 9 years, as this age range is important because most children have their four first permanent molars erupted. This is an optimal period for MIH screening and early diagnosis, limiting the amount of time that PEB and carious lesion development can occur in older cohorts, that may confound MIH diagnosis (Allazzam et al. 2014; Ghanim et al. 2011). Although the prevalence of MIH can increase or decrease depending on the year of birth (Koch et al. 1987), and as the aetiology of MIH is still uncertain, we cannot conclude there is a close relationship between age cohorts and MIH characteristics.

In the present study, bivariate analyses indicated a correlation trend, albeit statistically insignificant putatively due to the relatively small sample size, between childhood disease in the first three years-of-life and the use of antibiotics in the same period and MIH. The influence of early life health factors on MIH remains contentious. A 2022 meta-analysis by Garot and colleagues identified perinatal hypoxia, caesarean birth, and prematurity as major risks; postnatal conditions such as measles, otitis media, pneumonia, asthma and urinary tract infections were also associated with MIH (Garot et al. 2022). In Mexico, the caesarean birth rate is around 45% (Uribe-Leitz et al. 2019), significantly exceeding the 10—15% recommended by the World Health Organization (Organization 2021). In the present study, the prevalence was relatively lower at 34%; however, despite this high prevalence of caesarean deliveries there was not a statistically significant association between the mode of delivery and the presence of MIH, contrasting with a study of Garot and colleagues who reported a six-fold increase in MIH risk for infants with perinatal hypoxia and caesarean delivery (Garot et al. 2016). Moreover, in a retrospective parental questionnaire-based study of 1075 children from Medellin, Colombia, complications during the last trimester of pregnancy, low birth weight, respiratory problems, and urinary tract infections in the children were the factors most associated with MIH (Mejía et al. 2019).

In 2020, Lee and colleagues reported a relationship between MIH and maternal smoking during pregnancy and increased respiratory infections of the child in the first three years of life, putative causative factors for MIH (Lee et al. 2020). In the present study, only 1.4% of mothers reported smoking during pregnancy, and 30% of their children had MIH, a high prevalence but a very a small cohort that could be confounded by variables such as SES and reporting bias. The prevalence of smoking in Mexican women in urban areas is 18.4%, and there is a close relationship with high SES, and high SES in the present study was a protective factor for MIH, highlighting the potential for a confounding effect of multiple factors (Kuri-Morales et al. 2006; Ng et al. 2014; Ortiz-Hernández et al. 2015).

Current evidence suggests MIH results from a complex interplay of genetic, epigenetic and systemic factors, however, no single aetiological factor or group of factors has been conclusively identified (Garot et al. 2022). Retrospective studies, currently the dominant research model for investigations into aetiological studies of MIH, mostly rely on parental recall, which is prone to inaccuracy for events occurring at least six years prior. This is a limitation of the present study, as the search for an association between MIH presence and medical factors of the child or mother may have a high probability of recall bias. If data are sourced from health records, there is a possibility that the information collected is variable and not sufficient, lacking focus and consistency in recording data relevant to MIH. Meanwhile, longitudinal studies face high dropout rates and long-term feasibility challenges especially due to dropouts, with participants potentially being in the study from the last trimester of the mother’s pregnancy until the child is at least 6 years-of-age (for the eruption of FPMs and incisors) (Desai 2020). With MIH prevalence being high worldwide, its early diagnosis and management must be a priority in daily dental practice. The rates of hypersensitivity, enamel breakdown and early-stage caries in MIH patients underscores the importance of preventive strategies to improve outcomes. The present study contributes to the growing body of evidence highlighting MIH prevalence, clinical characteristics and risk factors, but many unanswered questions remain. Future research should focus on longitudinal studies with robust participant monitoring to ensure the highest level of accuracy. The challenges for MIH research and clinical management continue.

## Conclusion

MIH affected 12.3% of the population studied, with anterior teeth involved in 52% and hypersensitivity of at least one FPM reported by 30.6% of MIH-affected children. Demarcated lesions without PEB were the most prevalent defect, with white/creamy opacities occurring more frequently that yellow or brown lesions, and lesion severity increased with increasing number of affected FPMs. The factors associated with socioeconomic position such as father’s education, familial structure and presence or absence of siblings were related to MIH prevalence and severity.

## Data Availability

No datasets were generated or analysed during the current study.
